# The Almoners' Council and Patients' Payments

**Published:** 1921-04-23

**Authors:** 


### The Almoners' Council and Patients' Payments.

### AN INTERESTING REPORT.

The latest report of the Hospital Almoners' Council could hardly be shorter or more interesting. Without a superfluous word much information can be given in a little space. The four pages of the report itself are full of points, very pithily put, and Mrs. James Thomas, the Hon. Secretary to the Council, should presumably have the honour due to its draughtsman. The argument for the thorough training of an almoner is well put: her "work implies very considerable interference in the lives of others, and no one ought to intervene in such vital matters without having been thoroughly trained . . . so that, at all events, she is equipped with the experience and knowledge of the success and failure of social workers in the past." That is true, because while the failures of the amateur are glaring, the failures of the officious official have equally to be forsworn. She has to learn, we are reminded, that "the greatest asset in a man's own troubles is the man himself," and that, if help is needed, "it should be given adequately," to prevent as far as possible any recurrence. The almoner "will learn," also, "that hospitals, like other charities, have their limitations, and that these limitations must be recognised." We agree, and only wish that space had been found in the report for a summary of them.

The demand for trained almoners is said to be growing, and since the previous report almoners trained by the Council have been appointed to six London hospitals, and assistant almoners to four more, besides one to the Norfolk and Norwich Hospital. This provincial appointment emphasises the value of the Council's scheme to train candidates who intend to work outside London partly in the provinces themselves. Arrangements have been made for Part I. of the training of such candidates to be given at Glasgow, Leeds, and Liverpool Universities, and at Armstrong College, Newcastle.

It is remarked that, in addition to the high costs of maintenance and the lower purchasing power of money, hospital finance has to bear the burden of the greater number of patients which publicity in regard to public health proposals is bringing to their doors, while the special departments have similarly grown in size and in number. Hence the plan for contributions from patients. While the Council recognises the value to hospital budgets of a fixed sum whereof the yield is predicable, it favours the alternative plan of a contribution appropriate to each patient. Those to whom the flat rate is a burden may, it is argued, be discouraged from seeking treatment, while those who can afford more than the flat rate may shelter behind this and give to the hospitals less than they should. The Council therefore favours the second method of "inviting voluntary contributions from patients according to their means through the direct medium of the almoner." The report confesses, however, that "to justify an almoners' department from a financial point of view is not altogether easy," and a good deal might be said upon the other side. Nevertheless the report itself should be studied, for the argument is well put, though perhaps insufficient emphasis is laid upon the need for the almoner to work in close accord with the general policy which must emanate from the superintendent's office. The report concludes with a reprint of the article recently published in The Hospital upon "The Views and Aims of the Hospital Almoners' Council."

